# Expression of therapeutic misconception amongst Egyptians: a qualitative pilot study

**DOI:** 10.1186/1472-6939-10-7

**Published:** 2009-06-30

**Authors:** Mayyada Wazaify, Susan S Khalil, Henry J Silverman

**Affiliations:** 1 Department of Biopharmaceutics and Clinical Pharmacy, University of Jordan, Amman, Jordan; 2 Department of Obstetrics and Gynecology, Staten Island University Hospital, Staten Island, New York, USA; 3 Department of Medicine, University of Maryland School of Medicine, Baltimore, Maryland, USA

## Abstract

**Background:**

Studies have shown that research participants fail to appreciate the difference between research and medical care, labeling such phenomenon as a "therapeutic misconception" (TM). Since research activity involving human participants is increasing in the Middle East, qualitative research investigating aspects of TM is warranted. Our objective was to assess for the existence of therapeutic misconception amongst Egyptians.

**Methods:**

*Study Tool: *We developed a semi-structured interview guide to elicit the knowledge, attitudes, and perspectives of Egyptians regarding medical research.

*Setting: *We recruited individuals from the outpatient settings (public and private) at Ain Shams University in Cairo, Egypt.

*Analysis: *Interviews were taped, transcribed, and translated. We analyzed the content of the transcribed text to identify the presence of a TM, defined in one of two ways: TM_1 _= inaccurate beliefs about how individualized care can be compromised by the procedures in the research and TM_2 _= inaccurate appraisal of benefit obtained from the research study.

**Results:**

Our findings showed that a majority of participants (11/15) expressed inaccurate beliefs regarding the degree with which individualized care will be maintained in the research setting (TM_1_) and a smaller number of participants (5/15) manifested an unreasonable belief in the likelihood of benefits to be obtained from a research study (TM_2_). A total of 12 of the 15 participants were judged to have expressed a TM on either one of these bases.

**Conclusion:**

The presence of TM is not uncommon amongst Egyptian individuals. We recommend further qualitative studies investigating aspects of TM involving a larger sample size distinguished by different types of illnesses and socio-economic variables, as well as those who have and have not participated in clinical research.

## Background

For more than two decades, commentators have expressed concerns about research participants not distinguishing between research and clinical care [1-3]. Indeed, several empirical studies have shown that individuals participating in clinical research misconstrue a therapeutic intention to the research procedures in a study [4-10]. The term "therapeutic misconception" (TM) is used to describe this phenomenon, a term first reported by Appelbaum and colleagues in 1982 during interviews with patients with psychiatric disorders who participated in clinical trials [1].

At its basic level, TM involves a research participant's failure to recognize the distinction between the purposes of research and clinical care. Investigators have identified two ways in which TM can be manifested: 1) when research participants fail to recognize that decisions regarding randomization or certain aspects of the research procedures (e.g., dosages and duration of administered drugs) will not be individualized to their personal needs (TM_1_); or 2) when research participants hold an unreasonable appraisal of the nature or likelihood of medical benefit from their study participation (TM_2_) [4]. Essentially, research participants might hold mistaken beliefs about how the research will be executed (TM_1_) or is designed in a manner to ensure direct benefits to them (TM_2_) [1].

The presence of TM might be explained by participants' knowledge gap regarding how clinical research and patient care differ in their purpose, characteristic methods, and justification of risks [3]. Alternatively, it might be due to participants' misplaced trust in researchers, thinking that they will act as their physicians who will protect them, as well as promote their individual health interests [11]. Accordingly, participants might believe that investigators would not suggest enrollment unless it was very likely that they would benefit from the study or that the risks of research participation are without any substantial degree of risk. Regardless of underlying mechanisms, ethical concerns with the presence of a therapeutic misconception include the validity of informed consent (due to false beliefs regarding the magnitude of risks and potential benefits) and exploitation leading to inappropriate enrollment of patients into research.

Investigators have explored the frequency and the factors (both participant- and study-related) that might underlie the existence of TM in clinical research[4,6,7]. The identification of such factors, as well as attempts to prevent or mitigate the TM might have limited applicability to individuals in resource-limited countries, due to the existence of extreme poverty and lack of access to health care, high illiteracy rates, language and cultural barriers, gender inequality, and patients' enhanced unquestioning of their physicians' trust compared with that in the Western world. A few studies investigating the TM have occurred in the African setting [8-10]. The aim of this study was to assess the feasibility of investigating for the presence of TM in an interview study involving Egyptians attending public and private clinics. While our study is exploratory, we expect that our findings and experience will help towards the design of future TM studies in this type of population.

## Methods

### Sample and Setting

This study used the content of the interviews obtained from a previous pilot qualitative study exploring the knowledge, attitudes, and perspectives of Egyptians regarding medical research [12]. This previous study had recruited a convenient sample of 15 individuals from the outpatient clinic waiting areas from two of Ain Shams University hospitals: Ain Shams University Specialty Hospital, a semi-private university-based hospital; and Ain Shams University Public Hospital, a public teaching hospital. Private hospitals in Egypt serve patients with a higher socio-economic status than the public clinics. Both hospitals are situated in the metropolitan area of Cairo and serve as referral hospitals for patients predominantly coming from northern (Lower) Egypt as opposed to southern (Upper) Egypt. Lower Egypt consists of Cairo and areas of the Nile Delta, which fans out north of Cairo to the Mediterranean coastline to include Alexandria and Port Said. Urban life in Lower Egypt is marked by some 20–30 percent of the population living below the poverty line and an illiteracy rate of approximately 55 percent. Upper Egypt is predominantly rural and extends from 120 kilometers south of Cairo to the border with Sudan. One third of the population and half of Egypt's poor live in Upper Egypt and it is also the area with the highest infant mortality rates, 36% above the national average [13].

### Recruitment Process

Participants were recruited from the clinics' waiting rooms; they were either patients waiting for their clinic appointments or members of the lay public accompanying family members who had clinic appointments.

### Semi-structured interview tool

The survey tool consisted of open-ended questions that assessed their knowledge, attitudes, and perceptions regarding the following:

• clinical research

• their willingness to participate in different types of research, assessed by the use of short clinical research vignettes

• research concepts (e.g., randomization, double-blinded, equipoise)

• value of informed consent

• motivations of researchers

### Interviews

All interviews were conducted in colloquial Arabic. Interviews lasted approximately 45 minutes and were audiotaped. All of the interviews were conducted during a period of six months. Audio recordings were transcribed in Arabic and then translated into English. Interviewers helped participants complete a form that collected demographic information.

### Analysis

We performed a content analysis by having the three authors (MW, SSK and HJS) independently code the content of the transcripts based on the two categories of TM that had been previously identified (*a priori *coding) [14]. Each author scored the relevant responses dichotomously (0 or l) for the presence of TM_1 _and TM_2_, as defined above. All three authors discussed and compared the responses and differences were reconciled in iterative meetings [15]. In scoring for TM_2_, we made an effort to distinguish unreasonable appraisals in the extent of direct benefits from clinical research from two related but different concepts: 1) therapeutic misestimation, which involves misunderstanding the probability of direct benefit or harms that might result from research participation; and 2) therapeutic optimism, which involves participants hoping that they would be one of the lucky few who would receive a benefit [16].

### Confidentiality

Participants were assigned a unique name different from their actual name. These names were used during the interview. This unique name was coded to the individual's demographic form, which did not use the individual's real name. Tapes were destroyed after they were transcribed.

### Informed Consent

All participants gave their informed consent, which was given verbally, witnessed, and documented on the tape recording.

### Ethics Approval

The research ethics committees at Ain Shams University and the University of Maryland, School of Medicine gave approval for the conduct of this study and approved the mechanism of obtaining verbal consent.

## Results

### Demographic and background information

Of the 15 participants, 10 were female and 5 were male. Nine were recruited from the private hospital and six were recruited from the public hospital. Thirteen of the participants were from Lower Egypt, whereas two resided in Upper Egypt. The age range was from 19 to 69, with a median age of 30 and an average age of 38 years. Three participants had completed high school, eight had an undergraduate college degree, and four participants had attained a degree higher than undergraduate level. Of these participants, eight were patients waiting for their clinic appointments, while seven were accompanying family members to the clinic. Eight participants were married, all with children, while seven participants were single. All but one participant was unemployed; nine were considered to be in the lower income bracket (less than 1000 Egyptian pounds/month or less than $175/month total household income). For each participant who agreed to participate in the interview study, approximately four were approached. Common reasons given for refusal to participate in the study included time constraints or fear of missing their appointments.

### Manifestation of the Therapeutic Misconception (TM)

Our findings showed that a majority of the participants (11/15) expressed inaccurate beliefs regarding the degree with which individualized care will be maintained in the research setting (TM_1_). A smaller number of participants (5/15) manifested unreasonable beliefs in the nature or likelihood of the benefits that one could obtain in a research study (TM_2_). Of these 15 participants, 12 were judged to have expressed a TM on at least one of these bases, whereas 4 of the 15 of the participants expressed a TM on both of these bases.

#### Responses Reflected of TM_1_

Examples of responses that were scored as reflecting inaccurate beliefs that care would be individualized (TM_1_) included the following:

Interviewer: *"Do you think the medical care you get from a study-doctor will be the same type of care that you'll get from a doctor who isn't doing a study?*

Participant: *"No, there wouldn't be a difference"*

To a similar line of questioning, another participant had the following response (a combination of TM_1 _and TM_2_):

Interviewer: *Would you be concerned that they are more concerned with the research they're doing rather than providing individualized care?"*

Participant: "*No, because the goal of research is to make people better. They won't neglect their care, just because of the research"*

In contrast, one participant realized that his/her own physician would administer care that was more individualized than that received in a research study:

Interviewer: *"Do you think the medical care that you get from a study-doctor will be the same type of care you will get from a doctor who is not doing a study?"*

Participant: "*I think it would just be better care from someone that has known me for a long time."*

When asked regarding the prospect that the study-doctor will not know which drug would be administered to the patient (i.e., doctor being blinded) or that administration of the drug will be done by a random process, the following responses suggest there was disbelief that such a process would occur or they simply ignored what they were being told to them:

Interviewer: *"How would you feel if your doctor would not know which drug you would be taking?"*

Participant: *"There's no way that my doctor doesn't know what I'm taking"*

Interviewer: *"If a research study involves comparing a new drug with a current one, and your doctor does not know which drug is better for you. How do you feel about this?*

Participant: *"He'll probably try the new drug to see how it helps me"*.

Interviewer: "*If in a study there's a 50% chance of getting the old drug and a 50% chance of getting the new drug, where they'd determine which one you received in a random manner like the lottery, would you agree to participate?"*

Participant: "If I'll get better, I'll agree, why not?"

When asked regarding their thoughts about the concept of equipoise, there were expressions indicating that they would nevertheless receive the better drug:

Interviewer: *"Would it matter to you though, if you got the newer drug or the older drug, even though it's believed that they have the same risks and benefits?*

Participant: *"I think my physician would know better, and depending on their recommendation I'll take what they suggest"*

Interviewer: " What if the doctor doesn't know if the Urinex or the newer drug is better for you....?"

Participant: "*I'll take it if my doctor tells me that it's better"*

Interviewer: *"If it's the same study mentioned earlier, would it matter to you if you received the old drug or the new drug?"*

Participant: *"Yes it makes a big difference to me [which drug I take]*.

Interviewer: *"How so?"*

Participant: *"I'd prefer to take the old drug of course"*

#### Responses Reflecting TM_2_

The following are examples of responses indicating participants' unreasonable appraisals of the magnitude and likelihood of benefit that one obtains from participating in a research study:

Participant: *"If the study will have a negative impact on me, I won't participate. It must have some guidelines that guarantee that it will protect me, so that I'll agree to participate"*. Later in the same transcript:

Participant: *"It's like I said, you usually won't resort to entering a study or a [clinical] trial unless you feel like there's something that you may gain from being in it"*.

Interviewer: *"Would you agree to participate in a medical research study?"*

Participant: *"Certainly these studies won't have side effects, the goal is to improve health and not cause harm"*.

Interviewer: "*Would you allow one of your children to be in a research study?*

Participant: "*I must be at least 90% reassured that it's something safe*."

Essentially, participants believed that there was good assurance that clinical research was associated with benefits or that there would be no or relatively low risks, thus calling into question participants' understanding of the risk/benefit ratio associated with such studies.

## Discussion

The majority of the participants in this study manifested a misconception regarding how their personal care in medical research might be compromised by the procedures of the study (TM_1_). A smaller number also had a misconception of what is achievable in terms of benefits from the nature of the clinical trial (TM_2_). According to Appelbaum and colleagues, TM occurs "when a research subject fails to appreciate the distinction between the imperatives of clinical research and of ordinary treatment, and therefore inaccurately attributes therapeutic intent to research procedures" [1]. The National Bioethics Advisory Commission (NBAC) defined TM as "the belief that the purpose of a clinical trial is to benefit the individual patient rather than to gather data for the purpose of contributing to scientific knowledge" [17].

Commentators have hypothesized several conceptual foundations for the presence of TM. First, potential participants might harbor a misunderstanding of how clinical research and patient care differ in their purpose, characteristic methods, and justification of risks [3]. In addition to this knowledge gap, the existence of TM could be explained by the fact that research studies occur almost exclusively in the clinical setting, which might reinforce the presence of treatment relationships and individualized care. Essentially, presumptions acquired by patients in the clinical setting are brought with them to the research setting [18]. Accordingly, many patients might think that their care will still be individualized to match their personal medical needs even in the context of a research study. Such perceptions might be enhanced by the use of certain language found in consent forms (such as 'doctor' instead of investigator', and 'treatment' instead of 'intervention') or when patients are recruited into clinical trials by investigators who are also their primary physicians (although one study found that participants recruited by their physicians were not more likely to exhibit TM than others [7]).

Alternatively, many aspects of research participation might be construed as clinical activities and therefore, participants might see research as just another way of providing care for them [19]. For example, research activities that include close monitoring for symptoms and side effects, health education and psychosocial support provided by members of the research team, and the prescribing of medications are similar to the activities that occur in the clinical setting. It is therefore not surprising that one study found that many participants saw researchers as providing better patient care than healthcare providers [19]. Finally, the misconception may be related to emotions that participants have towards researchers, with consequent thinking that researchers will act as their physicians who will protect them as well as promote their individual health interests [20]. Accordingly, there might be concerns about misplaced trust towards researchers and researchers' exploitation of such trust [11].

Our study, being exploratory in nature, used a semi-structured interview technique that involved both patients and members of the lay public and relied on presenting hypothetical clinical research scenarios to these participants. Other empirical studies have used a variety of approaches to assess for the presence of TM. For example, studies have explored this phenomenon in patients who have participated in clinical trials [4-7,21] or have asked individuals to respond to different types of hypothetical clinical research scenarios [8-10]. These studies have also assessed for the presence of TM in different types of clinical trials (e.g., phase 1, 2 or 3 trials) or in different patient populations (e.g., those with cancer, psychiatric illnesses, heart disease, and inherited diseases). Finally, studies have used different methodologies to measure for the presence of TM, which have included the use of focus groups sessions [8], semi-structured interviews [4,7], survey [5], or forced responses to discrete questions [6,21].

We believe that qualitative research design methods are preferred when exploring the presence of and the explanations that can account for TM. Such studies can perform a more extensive probing into the reasons for the participants' TM. For example, qualitative studies can explore for how patients view care in the research setting, their feelings about the researchers juxtaposed upon those for their primary physicians, and for the presence and type of trust they place in researchers compared with their physicians. Also, commentators have claimed that a concept of therapeutic misconception should be distinguished from that of therapeutic misestimation, which occurs when participants understand the distinction between research and clinical care, but their skewed estimates of risks and benefits result other reasons, such as a misinterpretation of probability information [16,22]. Quantitative methods would fail to uncover such distinctions. Also, use of a focus groups methodology, because it stimulates dialogue, might be more useful for participants who have not thought out in detail their beliefs regarding research participation. Finally, studies should focus separately on members of the lay public with and without prior research participation (using hypothetical scenarios) and patients participating in different study trial designs and with different types of illnesses (e.g., acute and chronic illnesses).

Several studies have investigated the existence of participant- or study-related factors associated with the presence of TM through multivariate analysis [4,6,7]. In a study involving patients in different types of clinical trials, those with greater age, lower levels of education, poorer health and functional status, and those with greater optimism about their health in the future were at higher risk for manifesting TM [4]. In another study involving early phase gene transfer trials, participants with cancer or vascular diseases had lower TM scores compared with those with inherited or infectious diseases. Also, TM scores were significantly lower in patients when they received a consistent message from the study teams and consent forms that benefits were unlikely, compared with those who did not receive any such messages [7].

There are two major ethical concerns with the TM. First, failure to appreciate correctly the risks and benefits of research participation raises concerns regarding the validity of informed consent [3,4,23]. Indeed, understanding is an important requirement of informed consent, which itself is fundamental to ethical clinical research. Second, the presence of TM reflects the very real possibility that research participants will see themselves as patients and trust researchers as if the investigator's role was that of the physician. The resulting concern is that patients will be susceptible to exploitation, as investigators might take advantage of such misplaced trust to enroll them in clinical research [11,24]. The specific concern is that patients will view an invitation to enroll in research as a professional recommendation that is intended to serve their individual treatment interests [23].

We recognize several limitations of our study. First, the extent with which our findings are applicable to other populations in Egypt or other countries in the Middle East is constrained by the small sample size and the selection bias associated with obtaining information from only those who agreed to participate in this interview study. Second, the education level of our participants was higher than the median level of Egyptians from other parts of the country, thus questioning the applicability of our results to other regions in Egypt. However, Appelbaum and colleagues [4] showed that lower educational levels were directly associated with the presence of TM and hence, Egyptians with lower educational levels might demonstrate a higher prevalence of TM compared with the sample in this study. Finally, our study population was heterogeneous, as it consisted of a mixture of patients and the lay public and with individuals with different disease types. However, our study was more concerned with assessing the feasibility of performing interviews of Egyptians regarding their viewpoints on clinical research. Overall, our findings require validation in a larger sample that includes populations distinguished by different types of illnesses and socio-economic variables, as well as those who have and have not participated in clinical research. The results of such studies can lead to targeted education efforts focused on the underlying reasons for the presence of TM. Several studies have shown the effectiveness of targeted educational efforts in improving the comprehension and decisional capacities of research participants [25-27], including a positive effect on TM in some groups [28].

## Conclusion

In conclusion, the presence of TM is not uncommon amongst Egyptian individuals. Participants held a misconception regarding how their personal care in medical research might be compromised by the procedures of the study (TM_1_), as well as a misconception of what is achievable in terms of benefits from the nature of the clinical trial (TM_2_). We recommend further qualitative studies (both focus group discussions and in-depth interviews) investigating aspects of TM involving a larger sample size consisting of participants with different illnesses and socio-economic variables, as well as those who have and have not participated in clinical research.

## Competing interests

The authors declare that they have no competing interests.

## Authors' contributions

MW made substantial intellectual contributions to this study, was involved in the conception and design of the study, analyzed the data, and wrote drafts of findings. SSK made substantial intellectual contributions to this study, was involved in the conception and design of the study, collected the data and helped with the analysis of the data. HJS served as senior author, conceived the project, supervised data collection process, analyzed the data, and reviewed and edited draft manuscripts. All authors read and approved the final manuscript

## About the Authors

MW is Associate Professor in the Department of Biopharmaceutics and Clinical Pharmacy, Faculty of Pharmacy, The University of Jordan, Amman, Jordan. SK is a resident in Obstetrics and Gynecology at Staten Island University Hospital, Staten Island, New York. She received her medical degree from Ain Shams University, Cairo, Egypt. HS is Professor of Medicine at the University of Maryland, School of Medicine, Baltimore, Maryland. He is the Principal Investigator of a Bioethics Career Development Training Grant from the Fogarty International Center, National Institutes of Health.

## Pre-publication history

The pre-publication history for this paper can be accessed here:


